# Effect of Twisted Fiber Anisotropy in Cardiac Tissue on Ablation with Pulsed Electric Fields

**DOI:** 10.1371/journal.pone.0152262

**Published:** 2016-04-21

**Authors:** Fei Xie, Christian W. Zemlin

**Affiliations:** 1 Department of Electrical and Computer Engineering, Old Dominion University, Norfolk, Virginia, United States of America; 2 Frank Reidy Research Center for Bioelectrics, Old Dominion University, Norfolk, Virginia, United States of America; Georgia State University, UNITED STATES

## Abstract

**Background:**

Ablation of cardiac tissue with pulsed electric fields is a promising alternative to current thermal ablation methods, and it critically depends on the electric field distribution in the heart.

**Methods:**

We developed a model that incorporates the twisted anisotropy of cardiac tissue and computed the electric field distribution in the tissue. We also performed experiments in rabbit ventricles to validate our model. We find that the model agrees well with the experimentally determined ablation volume if we assume that all tissue that is exposed to a field greater than 3 kV/cm is ablated. In our numerical analysis, we considered how tissue thickness, degree of anisotropy, and electrode configuration affect the geometry of the ablated volume. We considered two electrode configurations: two parallel needles inserted into the myocardium (“penetrating needles” configuration) and one circular electrode each on epi- and endocardium, opposing each other (“epi-endo” configuration).

**Results:**

For thick tissues (10 mm) and moderate anisotropy ratio (*a* = 2), we find that the geometry of the ablated volume is almost unaffected by twisted anisotropy, i.e. it is approximately translationally symmetric from epi- to endocardium, for both electrode configurations. Higher anisotropy ratio (*a* = 10) leads to substantial variation in ablation width across the wall; these variations were more pronounced for the penetrating needle configuration than for the epi-endo configuration.

For thinner tissues (4 mm, typical for human atria) and higher anisotropy ratio (*a* = 10), the epi-endo configuration yielded approximately translationally symmetric ablation volumes, while the penetrating electrodes configuration was much more sensitive to fiber twist.

**Conclusions:**

These results suggest that the epi-endo configuration will be reliable for ablation of atrial fibrillation, independently of fiber orientation, while the penetrating electrode configuration may experience problems when the fiber orientation is not consistent across the atrial wall.

## Introduction

Ablation of cardiac tissue is an important treatment of cardiac arrhythmias, especially atrial fibrillation [1, 2]. Currently, ablation is done by either heating tissue with RF currents or cooling it, but both thermal methods have important limitations, in particular high recurrence rates [3, 4], significant complication rates [5, 6], and long procedure times [7, 8]. Ablation via membrane permeabilization, induced by pulsed electric fields, is an interesting alternative that has successfully been used in tumors [9] and more recently also in the heart [10–13].

An interesting question is whether pulsed electric field ablation also produces lesions of a geometry that is more likely to avoid recurrence. In particular, it is desirable to have lesions that have consistent cross sections across the myocardial wall or at least avoid substantial reductions in lesion thickness in any layer.

Previous work suggests that ablation with pulsed electric fields is successful if the local electric field exceeds some critical value [14]. Therefore, to predict the geometry of the ablated volume, we develop a model that allows us to compute the electric field distribution inside the myocardium. Cardiac muscle has an intricate fiber organization, in which the fiber orientation changes from epi- to endocardium [15]. Since the electrical conductivity is different in axial and transversal directions, the conductivity tensor likewise exhibits twisted anisotropy. In our model, we also include Joule heating to assess the heat generation during ablation. We validate the model by comparing predicted to experimentally determined ablation volumes and identify the critical field needed for ablation of cardiac tissue for a particular set of shock parameters.

Using this validated model, we go on to compute ablation volumes for different wall thicknesses and different degrees of anisotropy, for two electrode configurations: one configuration in which two parallel needle electrodes are inserted into the myocardium (“penetrating needles”) and one in which two circular electrodes are placed in opposition on the endo- and epicardial surfaces (“epi-endo”). By analyzing the geometries of the ablation volumes, we are able to make recommendations as to which electrode configurations should be advantageous for ablation in different situations. We also assess the role of Joule heating for the shock parameters that we used in our experimental demonstration of ablation with pulsed electric fields.

## Materials and Methods

The IACUC of Old Dominion University reviewed and approved our animal protocol for the experiments on which we report (protocol number 13–017).

### Modeling framework

We used the COMSOL Multiphysics finite element analysis software that allows modeling and simulation of many physics-based systems, including the computation of electrical fields in complex-shaped materials with arbitrary electrode positions. The AC/DC and Joule Heating modules (version 4.3b) can be used to solve for the local electric field and temperature distribution when electric fields are applied.

The results of our simulations are only stationary electric field distributions and heat dissipation rates (no transients). We also do not include tissue microstructure and do not attempt to model the detailed mechanism of ablation (which would involve the creation of pores in the membrane and exchange of extra- and intracellular fluids). While these simplifications are severe, they are justified by the observation that ablated regions closely match regions that exceed a critical electrical field strength in related studies [14].

Still, pulse width and frequency (and pulse number) do affect the ablated volume. A recent study sheds some light on the role of frequency in cardiac tissue[13], showing that longer pulses and larger numbers of pulses increase the amount of ablated tissue in a way that is very similar to increasing the pulse amplitude. In other words, the simulations presented here would still apply, but the ablation threshold, which is estimated to be 3 kV/cm here, would be different.

### Model geometry and mesh generation

Cardiac muscle is organized in fibers, and it is anisotropic as a result. In particular, electrical conductivity is higher and impulse propagation is faster in fiber direction than transversally [16]. We call the electrical conductivity in longitudinal direction *σ*_*L*_ and the transversal conductivities $\sigma_{T_{y}}$ (in y-axis) and $\sigma_{T_{z}}$ (in z-axis).

In ventricular myocardium, the fibers are organized in sheets with consistent fiber orientation. Going from epi- to endocardium, the fiber angle changes substantially, by 80–135°, depending on the species and the location in the heart that is considered [17–19].

**Fig 1:**
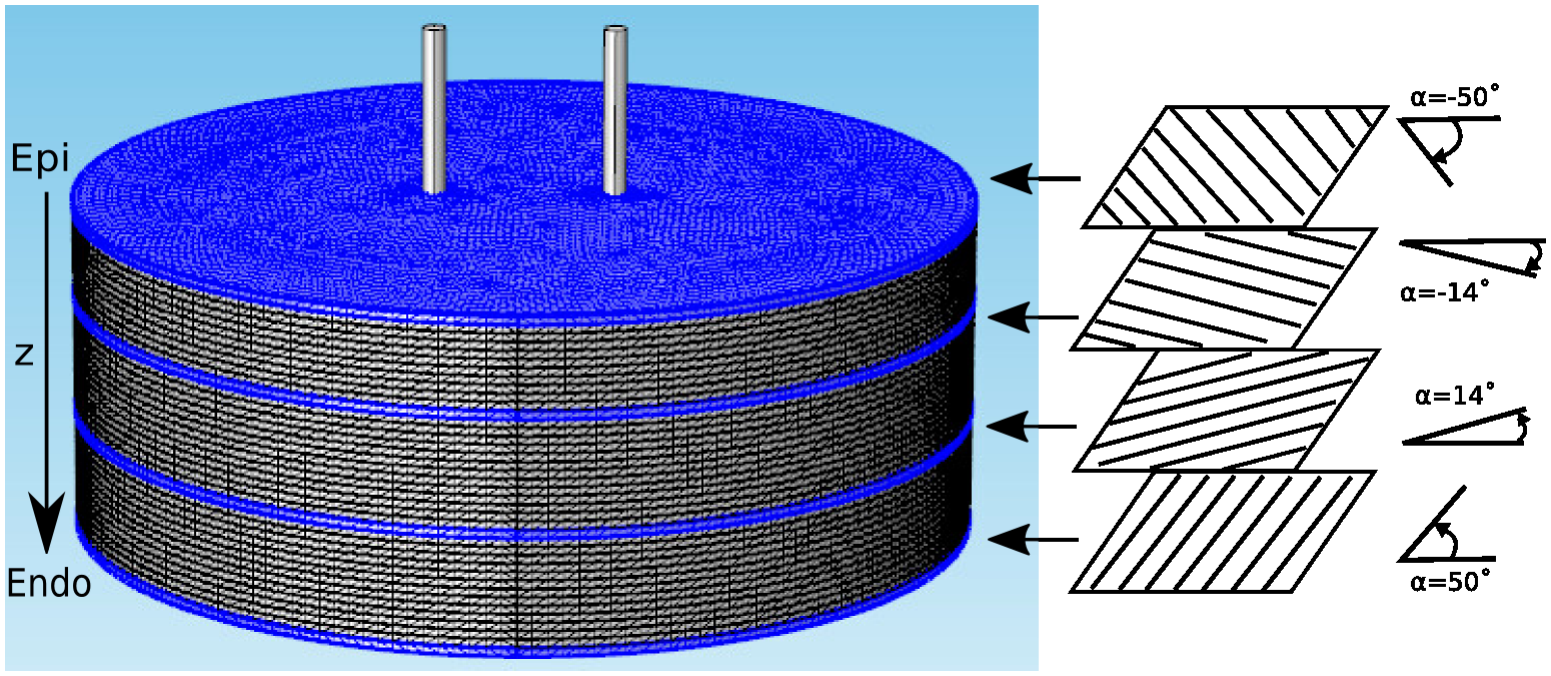
Geometry of our tissue model for the penetrating electrodes configuration. The tissue domain is cylindrical and the electrodes are placed symmetrically to the cylinder's axis. The tissue domain is discretized into tetrahedral elements (see Table 1 for details). Four of the 40 layers are marked blue (layers 0, 14, 25, and 39 from the epicardium), and the fiber orientation in these layers is illustrated and quantified on the right. The angle *α* is defined as the difference between the local fiber direction and the line through the points at which the electrodes intersect the layer.

**Fig 2:**
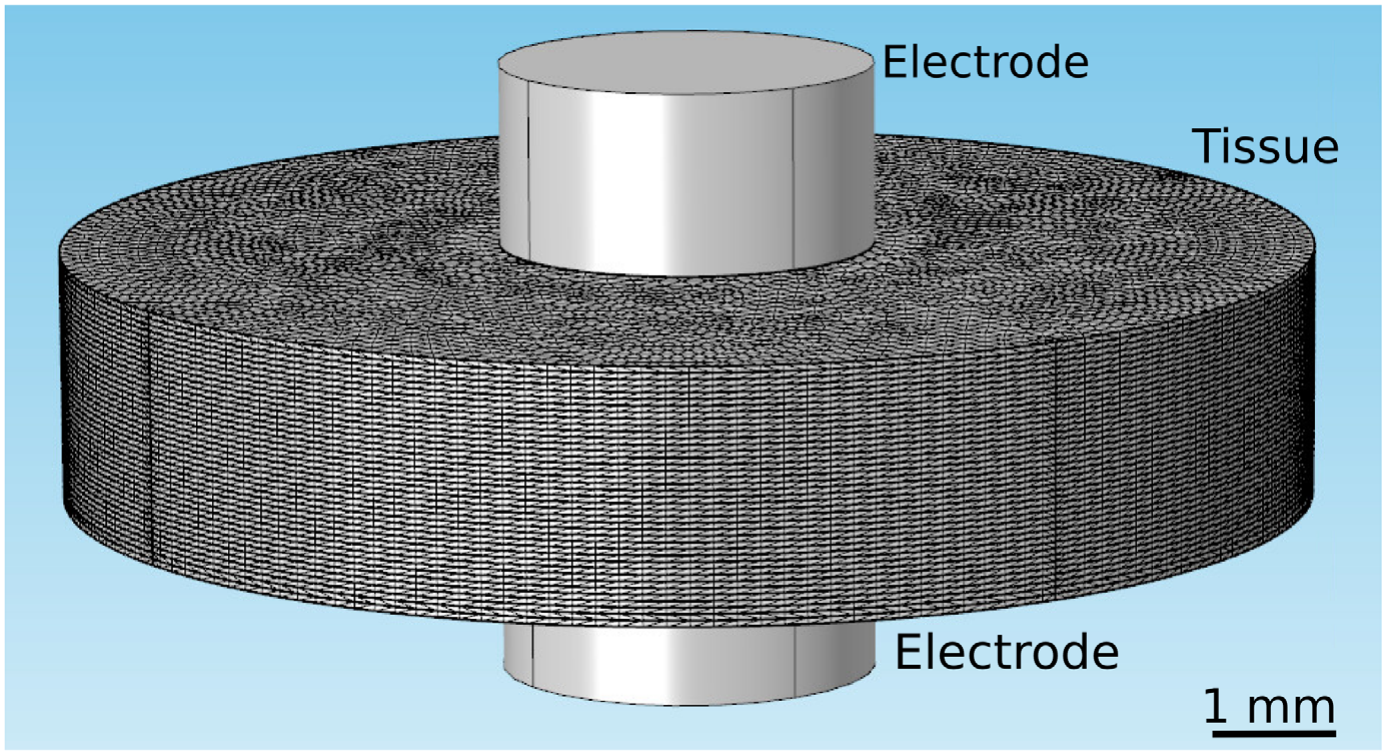
Geometry of our tissue model for the epi-endo electrode configuration. The tissue domain has the same dimensions as for the penetrating electrodes configuration (see Fig 2), but the electrodes are now cylinders placed in opposition to each other at the surfaces.

Using COMSOL, we constructed 3D models that replicate the geometries of experimentally relevant setups. In all cases, the tissue was a circular slab of 1–40 layers (see Fig 1). Anisotropy was implemented by assigning an anisotropic conductivity tensor to each layer, and the degree of anisotropy was quantified by the anisotropy ratio, *a* = *σ*_*L*_/*σ*_*T*_. Twisted anisotropy was implemented by rotating the fiber direction of the conductivity tensor from layer to layer (see Fig 1). We considered two electrode configurations: Two parallel penetrating electrodes (Fig 1) and two circular electrodes touching in opposing positions on epi- and endocardium (Fig 2). All simulations were performed all with several COMSOL-generated grids of increasing resolution. We chose the resolution for which further refinement did not lead to significant changes in the predicted ablated volume (we compared isosurfaces for different mesh resolutions in several sections and verified that the average isosurface shift was less than 100 μm).

We considered four different combinations of tissue thickness and anisotropy. Parameter set 1 uses a single layer (i.e. consistent fiber direction), and we use it to study the basic effects of anisotropy and the angle between the axis connecting the electrode positions and the fiber direction. Parameter set 2 describes a thin (4 mm) slab with low anisotropy ratio (*a* ≈ 2), with conductivities taken from a study that focused on rabbit ventricle [20]. These parameters are particularly relevant to our experimental validation, which was also done in rabbit ventricle. It should be pointed out that reported anisotropy ratios vary substantially both across species and within a species. The value *a* ≈ 2 is at the lower end of all reported values. It should not be construed as being representative of all rabbit tissue, but rather of low anisotropy ratio tissue, rabbit or otherwise. The helix angle *α* was varied from -50° to 50°, in accordance with an anatomical study in rabbits [18]. Second, we considered a thick slab (10 mm, typical of human tissue) with high anisotropy ratio (*a* = 10). As for parameter set 2, we do not claim that this anisotropy ratio describes all human tissue, but rather that it is an upper limit. Since for *a* = 10, we observed undesirable effects that may have clinical implications (see below), this high anisotropy ratio may be considered a “worst case” scenario. The helix angle *α* was varied from -75° to 60°, in accordance with an anatomical studies in humans [19,21,22]. Parameter set 4 considers a thin slab (3 mm) with high anisotropy ratio (*a* = 10), representative of human atrium. Large parts of the human atrium have consistent fiber orientation from epi- to endocardium [23, 24], and this situation is adequately described by parameter set 1. A new situation arises, however, when different fiber populations overlap, as it often happens in the left atrium, particularly at the junction of the pulmonary veins and the free atrial wall [25, 24]. In this situation, there are 2–3 layers of fibers with abrupt changes in fiber direction and a total fiber rotation of about 90°. In parameter set 4, we chose 3 layers oriented at 0°, 45°, and 90° (2 layers oriented at 0° and 90° give very similar results).

The electrodes were modeled as two intruding cylinders (penetrating electrodes case) or two discs touching the surfaces of the slab (endo-epi and endo-endo case). The full specifications of the models for the two electrode configurations are given in Table 1.

**Table 1:** Parameter sets used in numerical simulations.

	Parameter set 1	Parameter set 2	Parameter set 3	Parameter set 4
Description	Single layer	Thin / low anisotropy ratio	Thick / high anisotropy ratio	Thin / high anisotropy ratio
Electrodes radius and spacing for penetrating configuration	250 μm radius, 2 mm spacing	250 μm radius, 2 mm spacing	250 μm radius, 2 mm spacing	250 μm radius, 2 mm spacing
Electrodes radius and spacing for endo-epi configuration	N/A	3 mm radius, 4 mm spacing	2 mm radius, 10 mm spacing	2 mm radius, 3 mm spacing
Tissue dimensions	5 mm radius; 1mm thickness	5 mm radius; 4 mm thickness	10 mm radius; 10 mm thickness	5 mm radius; 3 mm thickness
COMSOL grid	4205 domain tetrahedral elements, 1013 triangular boundary elements (“normal” resolution)	Penetrating: 966,018 tetrahedral domain elements and 334,532 triangular boundary elements (“fine”); Epi-Endo: 788,547 domain elements and 282,858 boundary elements (“normal”)	Penetrating: 441,220 domain tetrahedral elements and 156,266 boundary triangular elements (“normal”); Epi-Endo: 828,018 domain elements and 284,970 boundary elements (“normal)	Penetrating: 84,237 domain tetrahedral elements and 10,500 boundary triangular elements (“fine”); Epi-Endo: 60,102 domain elements and 11,974 boundary elements (“extra fine”)
Tissue layers	1	40	40	3
Tissue fiber helix angle	Varied (0°, 30°, 60°, and 90°)	*α* = −50° (epicardium) to 50° (endocardium), each layer has the increment of 2.56° [18]	*α* = −75° (epicardium) to 60° (endocardium), each layer has the increment of 3.38° [19,21,22]	*α* = 0°, 45°, 90° [25]
Tissue conductivity	*σ*_*L*_ = 0.2 S/m, *σ*_*T*,*y*_ varied (from 0.02 S/m to 0.2 S/m)	*σ*_*L*_ = 0.264 S/m, *σ*_*T*,*y*_ = 0.126 S/m, *σ*_*T*,*z*_ = 0.217 S/m [20] (low anisotropy), *a =* 2.10	*σ*_*L*_ = 0.2 S/m, *σ*_*T*,*y*_ = 0.02 S/m, *σ*_*T*,*z*_ = 0.02 S/m [26] (high anisotropy), *a* = 10	*σ*_*L*_ = 0.2 S/m, *σ*_*T*,*y*_ = 0.02 S/m, *σ*_*T*,*z*_ = 0.02 S/m, (high anisotropy), *a =* 10

### Model equations

The electrical potential within the tissue associated with the pulse is determined by solving the governing Laplace equation for the electrical potential Φ:

−∇⋅(σ∇Φ) = 0. (1)

The boundary condition at the surface of the first shock electrodes is

$$\Phi \left(e_{1}\right)\;=\;V_{0},$$ (2)

while the electrical boundary condition at the surface of the second electrode is

$$\Phi \left(e_{2}\right)\;=\;0.$$ (3)

At any point in the tissue, the conductivity tensor can be written with respect to a properly oriented local coordinate system oriented as

$$\sigma_{0}=\left(\begin{array}{ccc} \sigma_{L} & 0 & 0\\ 0 & \sigma_{T,y} & 0\\ 0 & 0 & \sigma_{T,z} \end{array}\right)$$ (4)

To represent these conductivity tensors in one common coordinate system, we assume that the fiber angle *α* (see Fig 1) is a function of layer depth *z*, *α* = *α*(*z*), so *σ*_0_ from Eq 4 needs to be conjugated with a rotation matrix for *α*(*z*). The resulting conductivity tensor is:

$$\sigma \left(z\right)=\left(\begin{array}{ccc} cos^{2}\left(\alpha \left(z\right)\right)\sigma_{L}+sin^{2}\left(\alpha \left(z\right)\right)\sigma_{Ty} & cos\left(\alpha \left(z\right)\right)sin\left(\alpha \left(z\right)\right)\left(\sigma_{L}-\sigma_{T,y}\right) & 0\\ cos\left(\alpha \left(z\right)\right)sin\left(\alpha \left(z\right)\right)\left(\sigma_{L}-\sigma_{T,y}\right) & cos^{2}\left(\alpha \left(z\right)\right)\sigma_{T,y}+sin^{2}\left(\alpha \left(z\right)\right)\sigma_{L} & 0\\ 0 & 0 & \sigma_{T,z} \end{array}\right).$$ (5)

In two-dimensional simulations (parameter set 1), the conductivity tensor is simply

$$\sigma \left(z\right)=\left(\begin{array}{cc} cos^{2}\left(\alpha \right)\sigma_{L}+sin^{2}\left(\alpha \right)\sigma_{Ty} & \text{cos}\left(\alpha \right)\text{sin}\left(\alpha \right)\left(\sigma_{L}-\sigma_{T,y}\right)\\ \text{cos}\left(\alpha \right)\text{sin}\left(\alpha \right)\left(\sigma_{L}-\sigma_{T,y}\right) & cos^{2}\left(\alpha \right)\sigma_{T,y}+sin^{2}\left(\alpha \right)\sigma_{L} \end{array}\right).$$ (6)

All outer boundaries were assumed to be electrically insulating, i.e.

−n⋅J=0, (7)

where *J* is the current density.

The temperature distribution in the tissue can be obtained by solving a modified version of the Pennes bioheat equation with the inclusion of a Joule heating term:

$$\frac{\rho C\partial T}{\partial t}=\nabla \cdot \;\left(k\nabla T\right)+J\cdot E,$$ (8)

where *T* is the temperature. The tissue density *ρ* is chosen to be 1.06 g/cm^3^ [27], heat capacity *C* is chosen to be 977.16 J/(kg·K) [28], and the thermal conductivity *k* chosen to be 0.512 W/(m·K) [29].

The temperature dependence of conductivity was approximated by

$$\sigma \left(T\right)=\sigma_{0}\frac{1}{\left(1+\beta \left(T-T_{ref}\right)\right)},$$ (9)

Where *σ*_0_ is the reference conductivity (see Eq 4 and Table 1), *β* is resistivity temperature coefficient (we used 0.02/K), and *T*_*ref*_ is the reference temperature (we used 37.0°C). Using COMSOL, we solved the system of Eqs 1, 8 and 9.

### Quantification of lesion width variability across the wall

Our main concern is the consistency of lesion width across the wall. We used the ratio of the widths between the widest lesion in any one layer of tissue and the narrowest lesion in any one layer to quantify lesion width variability. Note, however, that the lesion thickness in any layer depends on the direction from which it is viewed so that the thickness ratio introduced above is really a function of orientation. We discuss this width variability function in more detail in the Results section (“3D geometry for twisted anisotropy”).

### Experiments

New Zealand white rabbits of either sex (3–4 kg, n = 12) were heparinized (500 IU/kg) and brought to a surgical plane of anesthesia with 2.5–4% isoflurane. The heart was rapidly removed, the aorta cannulated and flushed with ice cold Tyrode solution (in mM: NaCl: 128.2, NaCO_3_: 20, NaH_2_PO_4_: 1.2, MgCl_2_: 1.1, KCl: 4.7, CaCl_2_: 1.3, glucose: 11.1), and the heart was placed in a Langendorff-perfusion setup, where it was perfused and superfused with warm oxygenated Tyrode solution (37±0.5°C) at a constant pressure of 60–80 mmHg. After 30 min equilibration, 10–15 mM of 2,3-butanedione monoxime was added to eliminate contractions.

#### Ablation electrodes and choice of ablation sites

Ablation electrodes were made of two parallel 250 μm tungsten needles (see Fig 1). The electrode spacing was adjustable from 2 to 6 mm, and the electrodes were uninsulated over the terminal 4 mm and sharpened at the tip. Electrodes were dipped into surgical ink to mark the insertion sites and then inserted into the right or left epicardium, so that they penetrated the entire ventricular wall. Ablation sites were chosen at least 1 cm away from the septum.

#### Pulse generation

Nanosecond pulses were created with a transmission line generator. A double shielded coaxial cable (RG-217U) was used as a capacitor (C = 3.1 nF). An additional resistor *Z*_*m*_ = 13.7 Ω was placed in parallel with the heart to achieve impedance matching between the transmission line and the load. In theory, this setup should charge the transmission line until the breakdown voltage of the spark gap is reached and apply rectangular pulses of duration $t\;=\;\frac{2l}{v}$ to the load, where *l* is the length of the transmission line and *v* the speed of light in the transmission line. In our case, *l* = 35 m, *v* = 0.66*c* (*c* is the speed of light in vacuum), and consequently, *t* ≈ 350 ns [30]. The actual pulse shape was recorded with an oscilloscope (Tektronix 1001B, Beaverton, OR) and followed the theoretical prediction with good accuracy.

#### Ablation protocol

At each ablation site, we applied trains of 350 ns pulses (6 pulses at 3 Hz) of different amplitudes. We used an adjustable spark gap to allow the adjustment of the pulse amplitude, the pulse frequency was adjusted by adjusting the supply voltage, provided by a 0–20 kV power supply (EH Series, Glassman, Highbridge, NJ). When we generated trains of pulses, the pulse amplitude was reproducible within ± 0.2 kV over the amplitude range used in this study.

#### PI/TTC staining and sectioning

After the creation and electrophysiological evaluation, preparations were stained either with propidium iodide (PI, 30 mM/30 min) or tetrazolium chloride (TTC, 30 mM/20 min), for further study of the geometry of the ablated volume. For PI stains, we subsequently washed out the PI using our coronary perfusion for 40 minutes, leaving only the cells with compromised membrane stained with PI [31].

For both TTC and PI stains we cut lesions out of the tissue preparation and mounted each lesion separately in a block of agar. We then sectioned the lesions (section thickness: 300 μm). For PI stains, we recorded the fluorescence (illuminated at λ = 532 nm) for each section. By defining a fluorescence threshold that corresponds to dead tissue, the ablated area was identified.

## Results

### Anisotropy causes more complex field geometry

**Fig 3:**
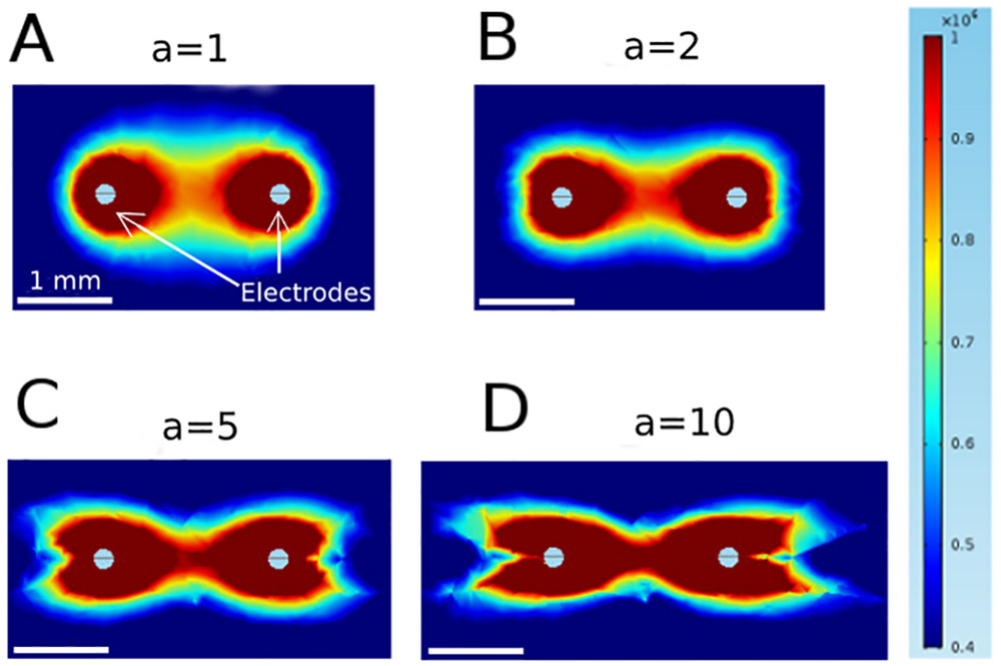
Contributions of the field components to the norm of the field for isotropic conditions (*a* = 1) and strongly anisotropic conditions (*a* = 10) using parameter set 1. The figure shows a zoom into the area around the electrodes, not the complete (circular) medium.

We started by considering the electrical field distribution for untwisted fibers for varying anisotropy ratios. Fig 3 shows the field distribution we computed for anisotropy ratios for an applied voltage of 2.3 kV. An anisotropy ratio of *a* = 1 (*σ*_*L*_ = *σ*_*T*,*y*_ = 0.2 S/m) leads to the familiar dipole field distribution (Panel A). Higher anisotropy ratios (achieved by varying *σ*_*T*,*y*_) do not simply stretch the graph of Panel A, but cause a flattening of the isofield lines at the left side of the left electrode and the right side of the right electrode (for *a* = 2, *σ*_*T*,*y*_ = 0.1 S/m), which develops into an indentation for *a* = 10 (*σ*_*T*,*y*_ = 0.02 S/m). Overall, the shapes of the isofield lines are considerably more complex for higher anisotropy ratios.

**Fig 4:**
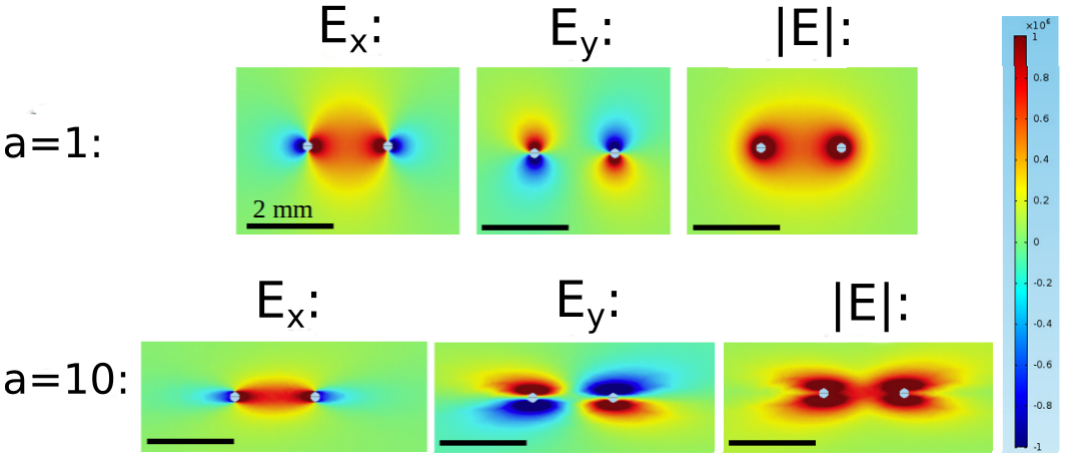
Effect of anisotropy on field distribution for penetrating electrode configuration (parameter set 1). They are obtained by varying the anisotropy ratio between 1 and 10, and keep the other parameters fixed. These simulations were carried out using parameter set I, but only with a single layer. The figure shows a zoom into the area around the electrodes, not the complete (circular) medium.

This higher complexity can be understood by considering the components of the electric field separately. Fig 4 shows these components along with the norm of the field, for both *a =* 1 and *a =* 10. It is worth noting, that even for *a =* 1, both *E*_*x*_ and *E*_*Y*_ have pronounced indentations: *E*_*x*_ is zero along the vertical line through each electrode and non-zero on either side, which *E*_*y*_ is zero along the horizontal line through both electrodes. In the norm $\left|E\right|\;=\;\sqrt{E_{x}^{2}+E_{y}^{2}}$, however, the components add up to create a smooth distribution. For *a* = 10, the field (and the currents, which are proportional to the field) become concentrated in the direct vicinity of the *y*-axis, and this also means that the *E*_*y*_-component has significant amplitude for a wider range of *x* values. Resistance in the *x-*direction becomes less significant and for current, traveling away from the target electrode (in *x-*direction) before traveling to towards it now adds less to the total resistivity of a path. This explains the complex geometry of the field for higher anisotropy.

### Field geometry depends on fiber angle

**Fig 5:**
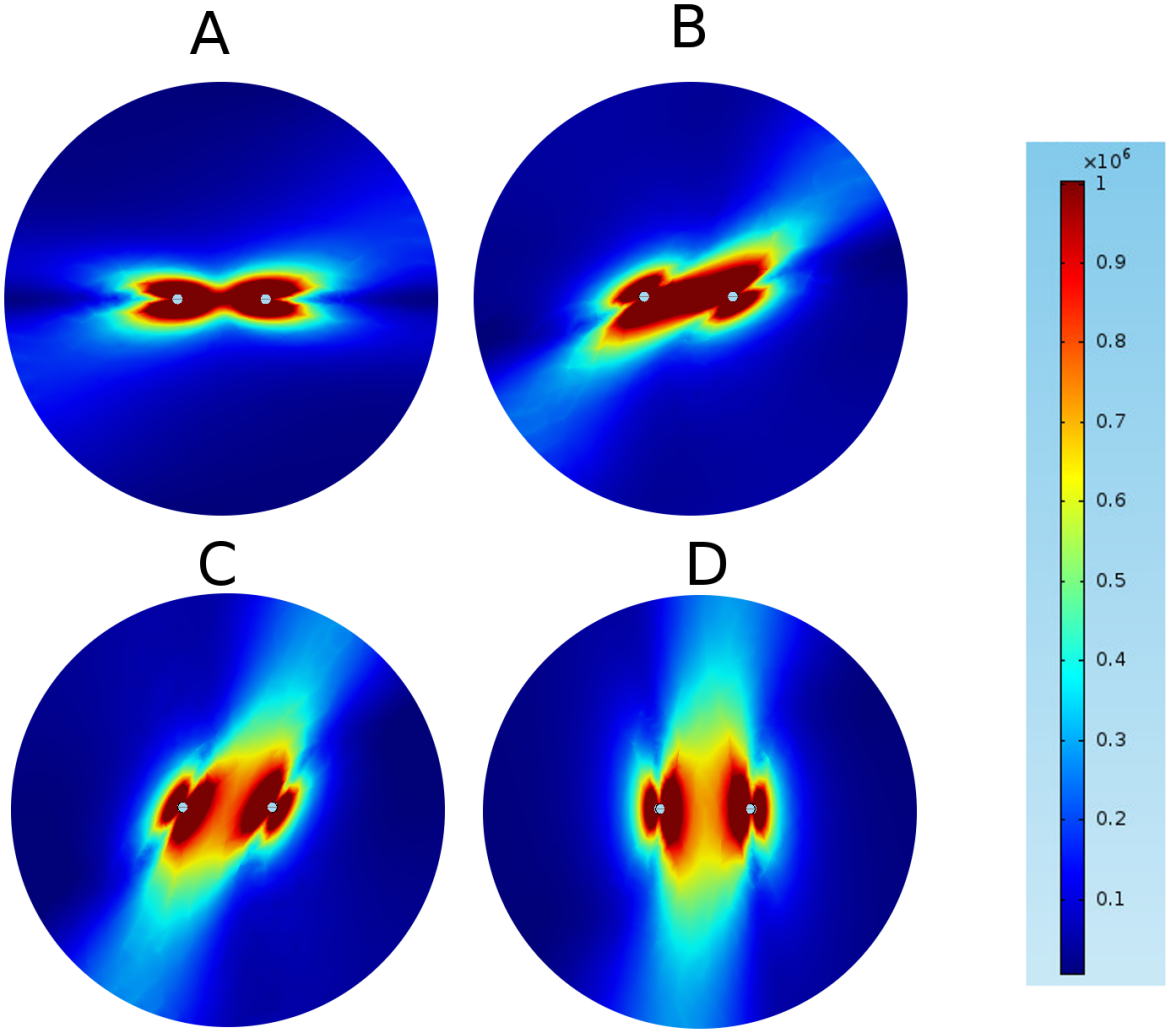
Effect of fiber angle on field distribution for strong anisotropy (*a* = 10) using parameter set 1. A: *α* = 0°. B: *α* = 30°. C: *α* = 60°. D: *α* = 90°.

Next, we consider the electrical field distribution for untwisted fibers of constant anisotropy ratio (*a* = 10) for varying fiber orientations. Panel A in Fig 5 (*α* = 0°) is identical to Panel D in Fig 4. Panel D in Fig 5 shows the distribution for vertical fibers (*α* = 90°), this distribution can be understood from Fig 4 with an argument similar to the one we gave for *α* = 0° above. Panels B and C in Fig 5 (*α* = 30° and 60°) follow the same logic, even if the fiber orientation off the coordinate axes leads to slightly more complex field distributions. The fiber orientation is evident in all field distributions as the direction in which the field is non-zero at the furthest distance.

### 3D geometry for twisted anisotropy: low anisotropy ratio (parameter set 2)

**Fig 6:**
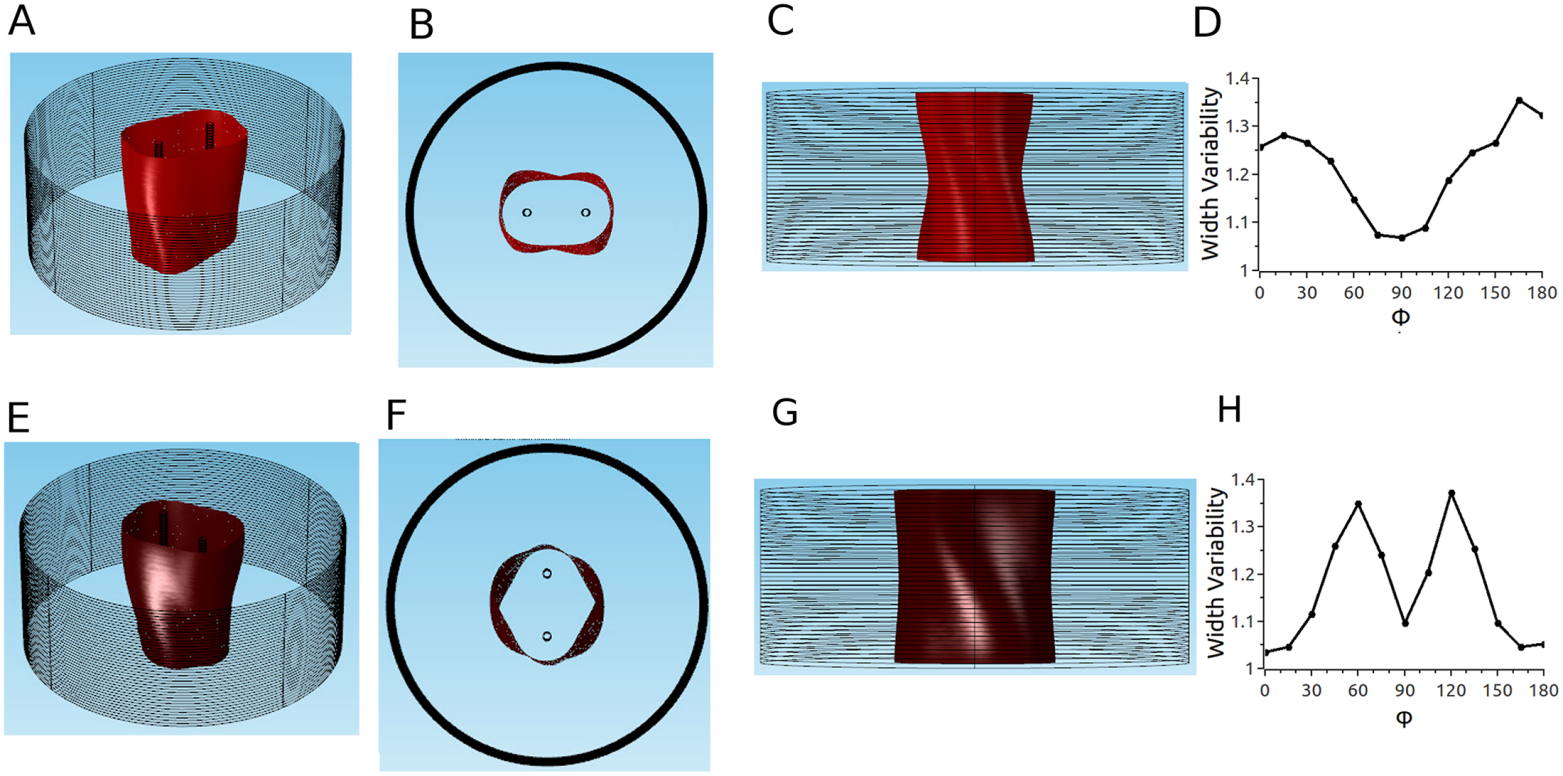
Predicted ablated volume for parameter set 2 and penetrating electrodes configuration. Red surface is the isosurface for |**E**| = 3kV/cm (applied voltage: 2.3 kV). A-D: Electrodes are oriented such that the line connecting them is in fiber direction. A: Oblique view. B: Top view. C: Side view. D: Width variability function. E-H: Electrodes are oriented such that the line connecting them is perpendicular to the fiber direction. E: Oblique view. F: Top view. G: Side view. H: Width variability function (see text and Methods section).

When we turned to twisted anisotropy, we first considered our parameter set 2 (*a* = 2, see Methods). Fig 6 suggests that for these conditions, the effects of anisotropy will be limited. Panels A-C show results for the case that the electrode axis matches the fiber direction, so that *α* varies from– 50° to 50° from epi- to endocardium. Panel B shows that the cross sections of the ablated volume are almost constant across the wall. Specifically, the lesion width (measured perpendicular to the line connecting the electrodes) in the center does not vary along the line connecting both electrodes. Panel C shows a projection in *x*-direction, which shows that there is modest variability in the lesion width from epi- to endocardium. Panel D quantifies this transmural width variability by dividing the largest width by the smallest width (for a given projection). The width variability depends on the projection direction. The projection in Panel C (Φ = 0) has a width variability of approximately 1.25, but for different projection angles Φ, the width variability varies between 1.05 and 1.4; this result makes sense if you carefully consider the 3D geometry of the lesion shown in Panel A. Panels E-H show corresponding results for electrode axis perpendicular to the fiber direction, so that *α* varies from– 5° to 95°. The cross sections vary a bit more in this case (Panel F), but are still moderate. The lesion width varies by less than 5% for Φ = 0 (Panel G), but the width varies in a range similar to the case of the electrode axis in fiber direction (Panel H).

### Critical field for ablation and comparison with experiment

It is reasonable to assume that in tissues that are homogeneous and exposed to the same temporal pulsing pattern, tissue survives if the field strength is below a certain threshold but is ablated if the field strength is above this threshold [14]. In cardiac tissue, it is not yet known what the critical field is.

**Fig 7:**
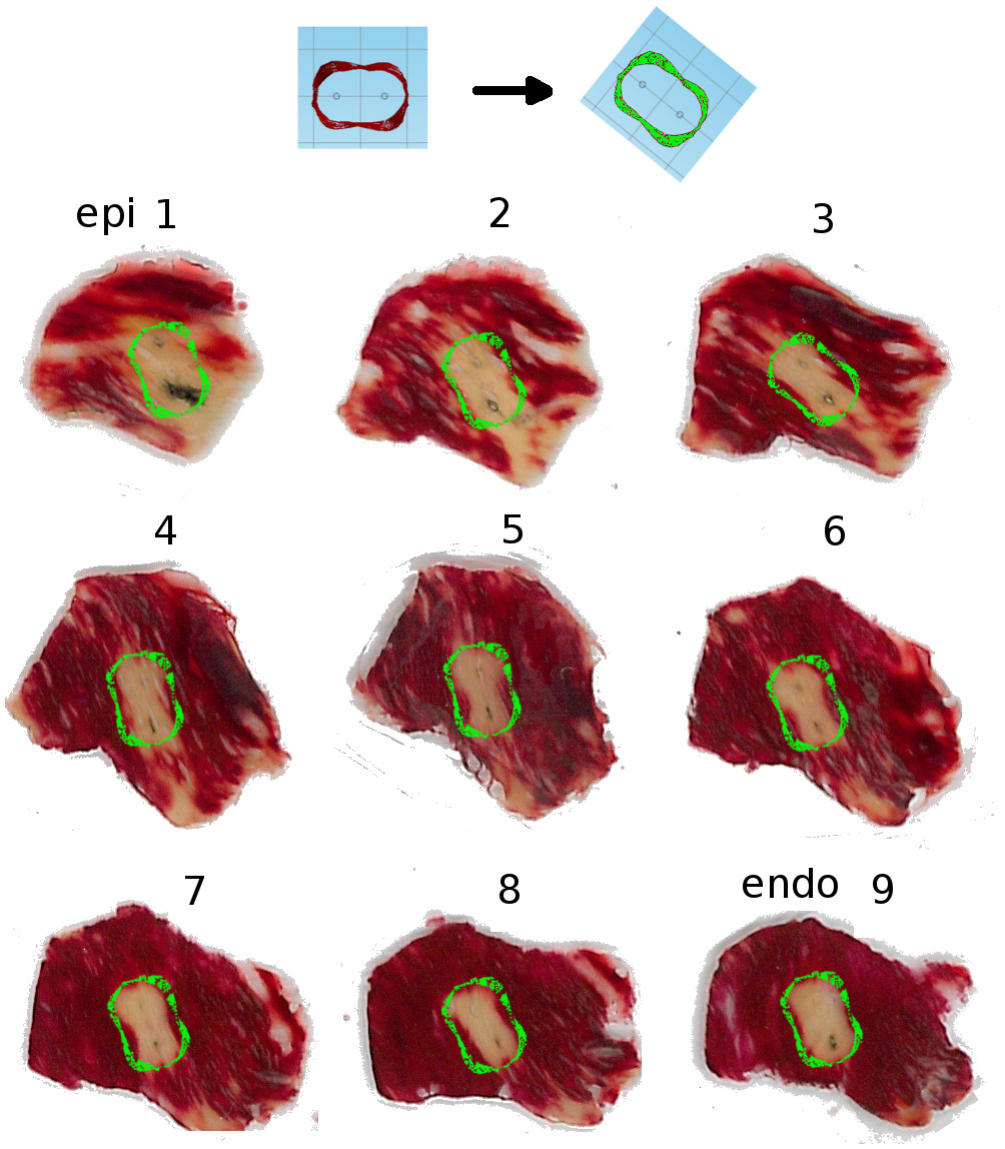
Comparison of predicted and experimentally determined ablation area for penetrating electrodes configuration. Panels 1–9 show sections of a lesion that has been stained with tetrazolium chloride (TTC), so that the ablated areas are white, while the surviving areas are stained red. We used the outline from Fig 6B as the theoretical prediction and oriented it so that the electrodes in the simulation match the electrodes in the experiment; the resulting outline is superimposed in green over each stained section.

We compared isosurfaces of different field strength with the outlines of the ablated tissue and noticed that the isosurface of 3 kV/cm produces a good match. Fig 7 shows all sections of a lesion, with the isoline for 3 kV/cm (respecting the electrode positions) is superimposed in every case. Note that there are some areas that were not stained by TTC even though they are far from the ablation site, especially in sections 1 and 2. We have observed such areas on the epicardium even in hearts that received no ablation treatment and attribute them to the limitations of our staining technique; however, the overall good fit gives us confidence that the 3 kV/cm isosurface will also give a good approximation of the ablated volume in other circumstances.

### Thermal effects

**Fig 8:**
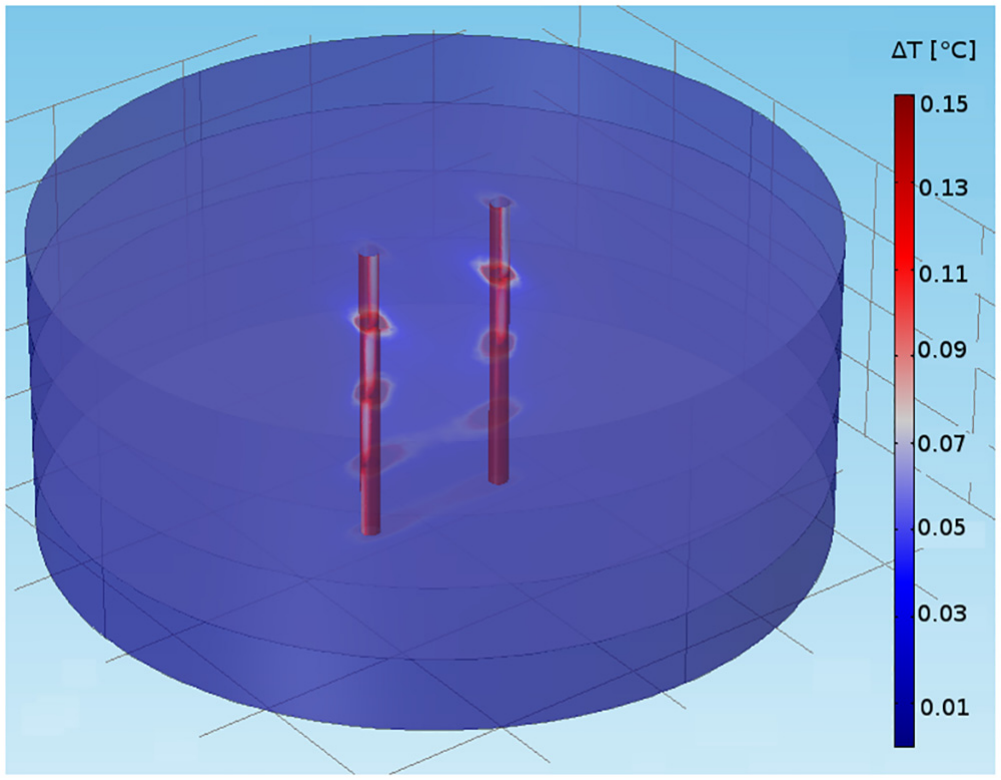
Thermal effects of pulsed electric field ablation. We show temperature distribution for a single shock (350 ns, 2.3 kV), for a medium with four layers with fiber orientations *α* = 0°, 30°, 60°, and 90°.

One of the major promises of pulsed electric field ablation is that it will be free from the thermal side effects that riddle RF ablation. Our simulated temperature distribution demonstrates impressively just how insignificant warming due to pulsed electric field application is (see Fig 8). Even at a distance of just 200 μm from the electrodes, the temperature increase after a single nanosecond pulse application is only 0.1°C, and it drops very quickly as the distance from the electrodes increases. This implies that shock protocols of 6–20 pulses, as they are used in our current experimental studies, will not lead to any thermal tissue damage outside the direct vicinity (200 μm) of the electrodes. In experiments we confirmed, both for single pulses and trains of 20 pulses (at 3 Hz) that heating was below 0.2°C at the midpoint between the penetrating electrodes.

### Endo-epi configuration

**Fig 9:**
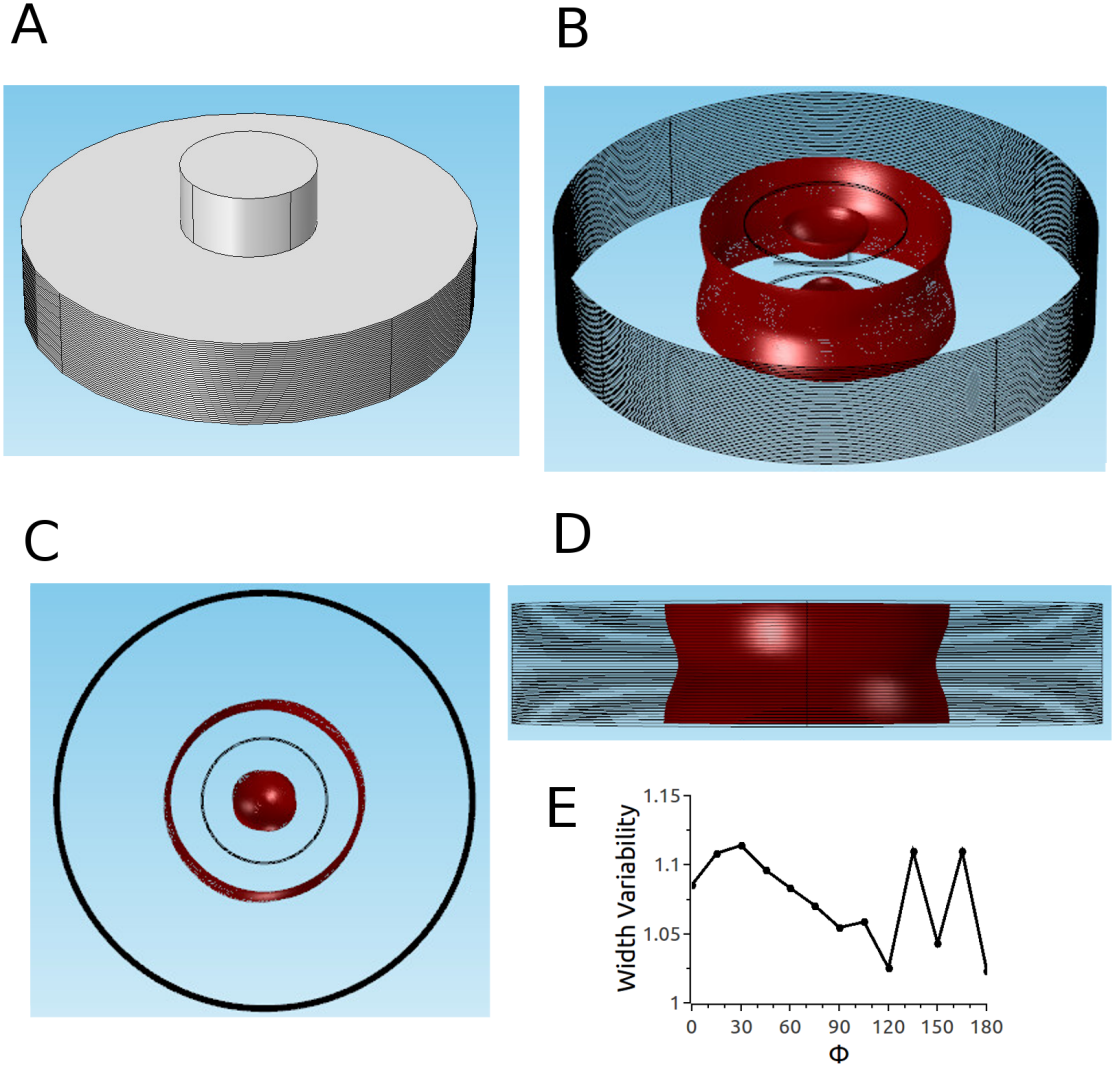
Predicted ablated volume for parameter set 2 and epi-endo electrode configuration. A: Epi-endo geometry. Red surface is isosurface for |E| = 3kV/cm. B: Oblique view. C: Top view. D: Side view. E: Width variability function.

We also considered a second electrode configuration (the medium is still described by parameter set 2), in which the electrodes do not penetrate the tissue but instead touch the epi- and the epicardium (see Figs 9A and 2). Fig 9B–9D show different views of the ablated volume. It is remarkable that even though the simulations incorporated twisted anisotropy, the predicted ablation volume is in very good approximation rotationally symmetric (Panel C). Furthermore, the cross section is close to constant for the chosen parameters (Panel D), so that the ablated volume is almost a cylinder. Panel E shows that the width variability changes only very little (from 1.02 to 1.12) with the projection angle.

### Thick tissue with high anisotropy ratio (parameter set 3)

Given the good match of model predictions (with parameter set 2) and experimental results for the case of rabbit ventricular tissue, it is interesting to study what predictions the model makes for the human cardiac tissue. For human ventricular tissue, we consider thicker tissue with a higher anisotropy ratio (parameter set 3).

**Fig 10:**
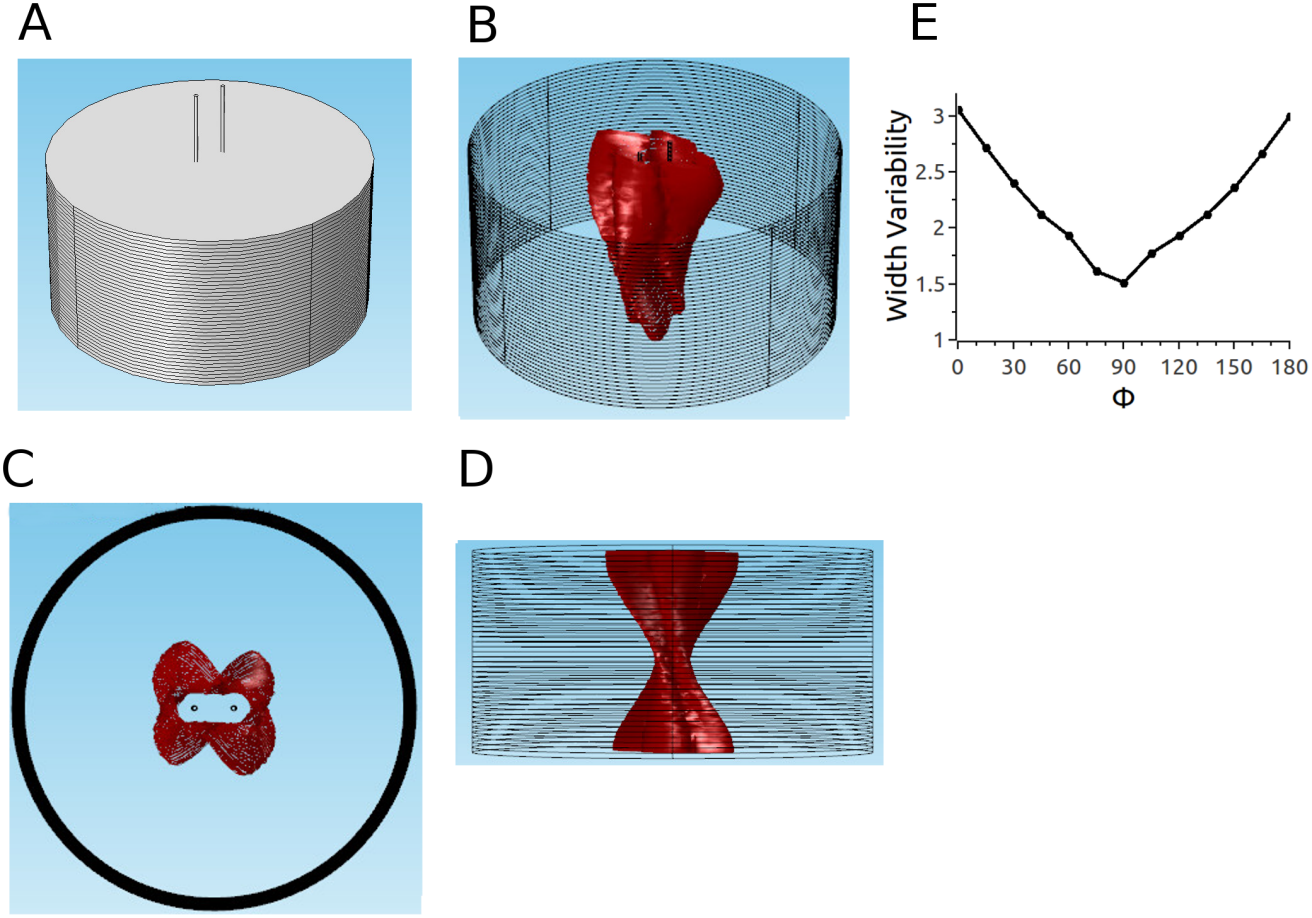
Predicted ablated volume for parameter set 3 with penetrating electrodes configuration. Details as in Fig 10. Amplitude of applied voltage is 2.3 kV.

Results for parameter set 3 are substantially different (Fig 10). The fiber direction and its twist are clearly evident in the 3D shape of the ablated volume (see Panel B). Indeed, the shape of the ablated volume is well approximated by taking the corresponding 2D ablated area for untwisted anisotropy (see Fig 3D) and moving it from epi- to endocardium while rotating it to keep it aligned with the fiber direction in all sections. Panel C, the top view, likewise illustrates the presence of elongated sections in different orientations (over the range of orientations present in the myocardium). In the projection with Φ = 0° (Panel D), it becomes apparent that this lesion geometry can be problematic if a uniform lesion thickness is desired. If multiple ablations would are performed and the electrodes are moved in the direction Φ = 0°, the result would be a lesion with the profile shown in Fig 10D. As shown in Panel E, the width variability for Φ = 0° is approximately 3, and while it decreases substantially towards Φ = 90°, its remains above 1.5 for all Φ.

**Fig 11:**
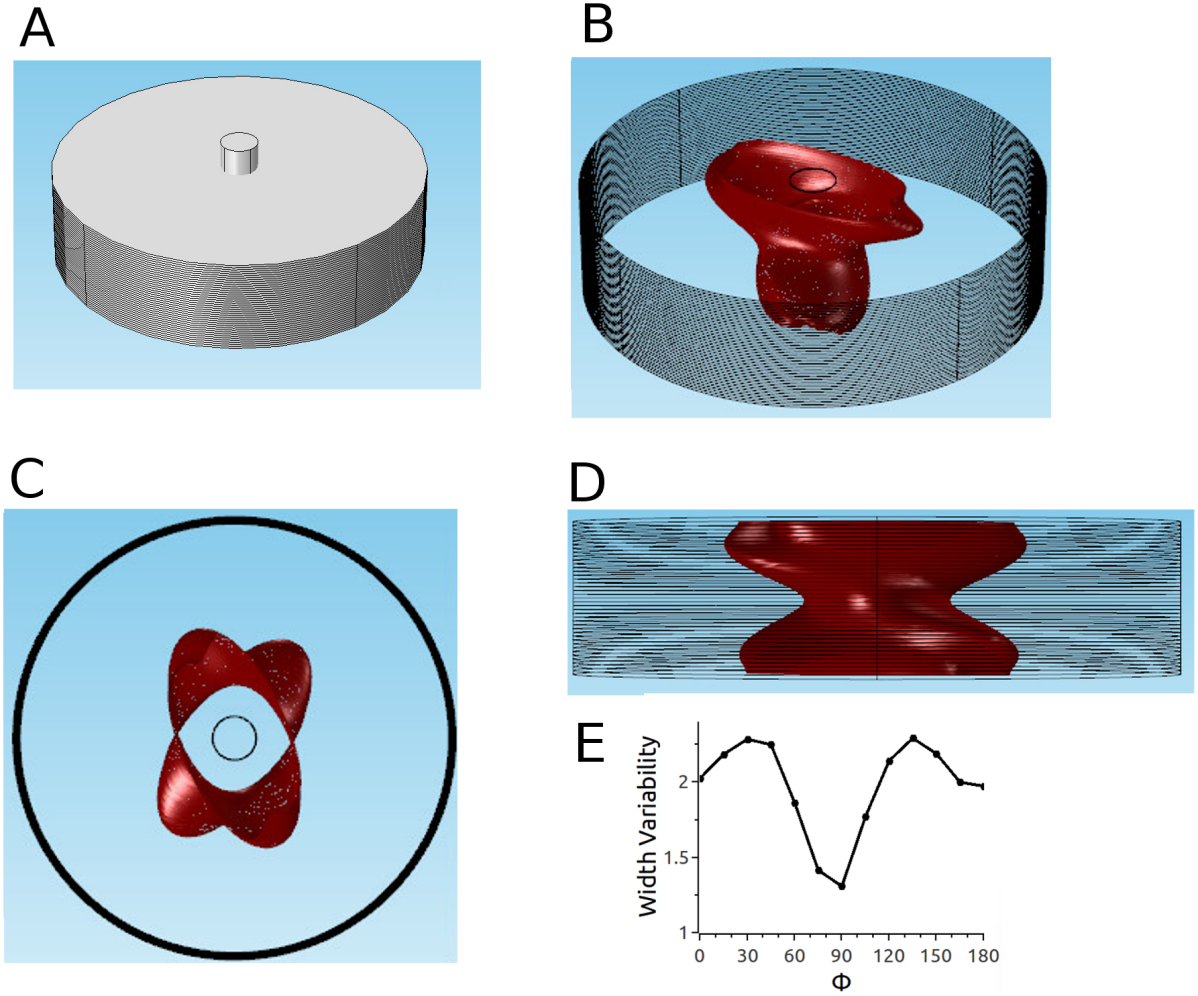
Predicted ablated volume for parameter set 3 and epi-endo configuration. Details as in Fig 10. Amplitude of applied voltage is 14 kV.

For the endo-epi configuration with parameter set 3, the predictions likewise change substantially (Fig 11). Again, the oblique view (Fig 11B) shows that the ablated volume is wider in the local fiber direction in every cross section. The top view (Fig 11C) shows two pronounced parts of the ablated volume, corresponding to the fiber directions at the epi- and the endocardium. These orientations stick out because currents in general fade off towards the center, which means that the red areas in each cross section are getting smaller towards the center. The side view has similarities with that for the penetrating electrodes configuration in that it leads to non-uniform lesion thickness (no matter with projection direction is considered). The quantitative comparison of the width variabilities (Panel E and Fig 11E) shows, however, that the endo-epi configuration produces more consistent lesion width (width variability range 1.3–2.3 vs. 1.5–3).

One qualitative difference to the penetrating electrodes configuration is that a minimum field is required to ablate a volume that reaches from epi- to endocardium (in the penetrating electrode case, there is a corresponding minimum field that is necessary to obtain a volume that connects both electrodes). While the threshold field in the penetrating electrodes case can be changed by adjusting the electrode separation, the threshold field for the epi-endo configuration is determined by the tissue thickness. This means that thicker tissues will require larger fields for transmural ablation, and lead to wider lesions, and if lesions beyond a certain width are not desired, the epi-endo configuration will not be appropriate for tissues beyond a certain thickness.

### Thin tissue with high anisotropy ratio and discrete jumps in fiber direction (parameter set 4)

**Fig 12:**
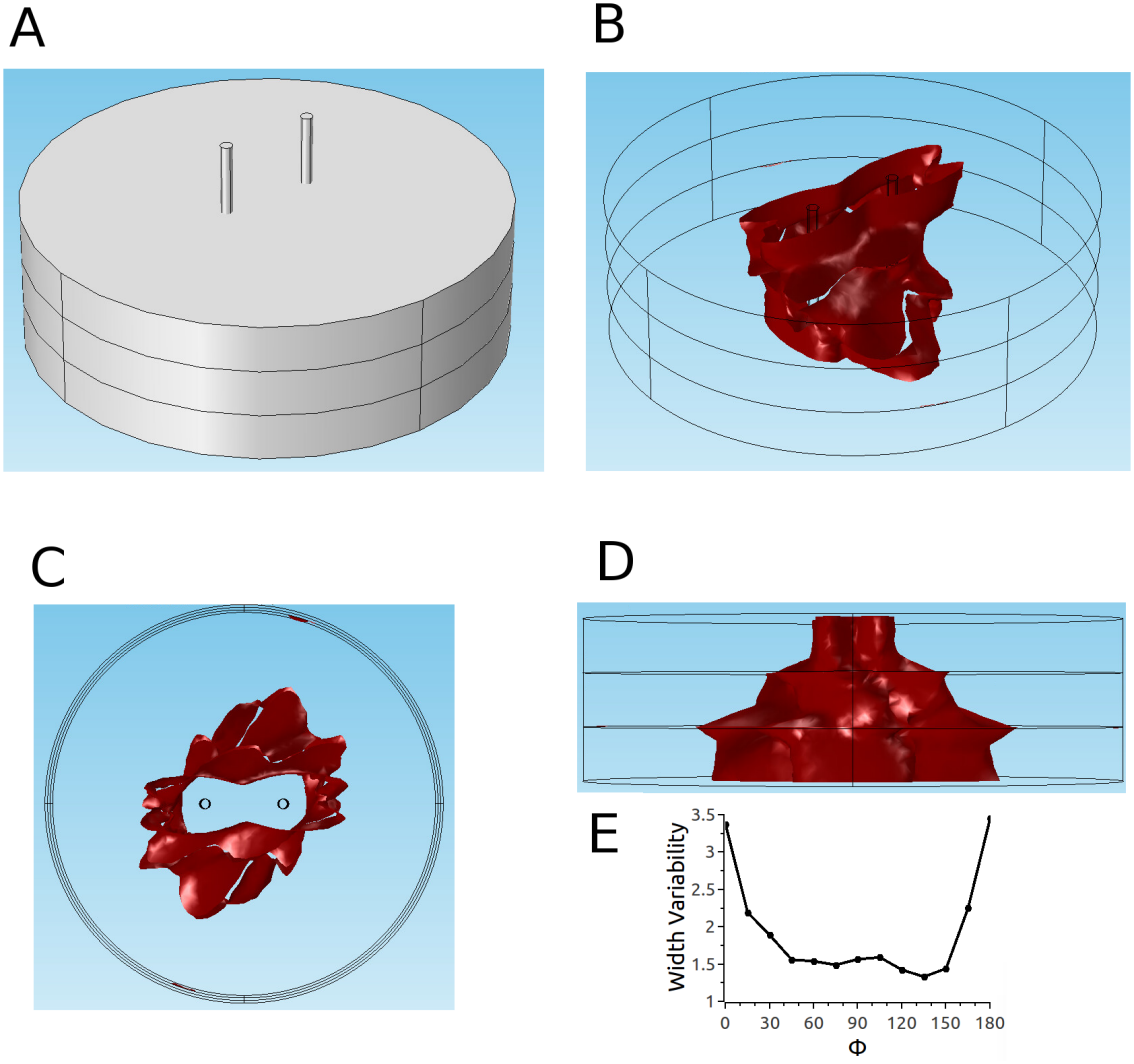
Predicted ablated volume for parameter set 4 and penetrating electrodes configuration. Details as in Fig 10. Amplitude of applied voltage is 1.8 kV.

The atrial myocardium does not have as many layers with different fiber directions as the ventricular myocardium; in fact, for large parts of the atrium, the fiber orientation is consistent from the epi and endocardium [25]. Only in certain regions, fiber bundles of different regions overlap, leading to 2–3 layers of different orientation from epi- to endocardium. The amount of fiber rotation can go up to 90°, and we modeled this situation with parameter set 4. In this case, the challenges of pulsed electric field ablation with the penetrating electrodes configuration are similar to the ventricular case, just more discrete: the cross sections of the ablated volume are oblong in every section, and the orientation of the cross sections follows the fiber direction (see Fig 12B and 12C). The side view (Fig 12D) shows that as in the human ventricular case (Fig 10D), there is a risk of producing lesions with very uneven width. This is quantified in Panel E, which shows that the width variability can go up to 3.3 (for Φ = 0°).

**Fig 13:**
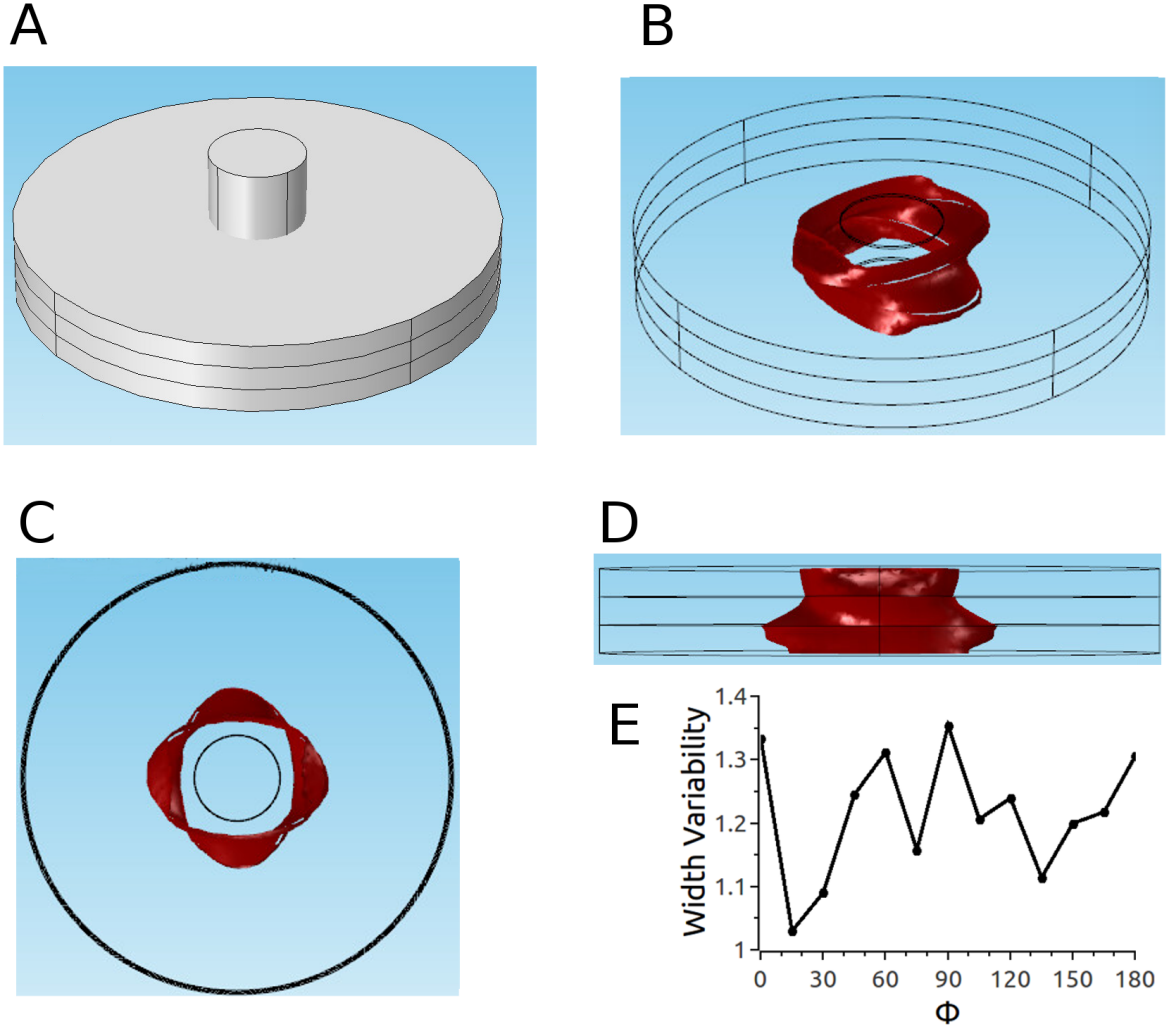
Predicted ablated volume for parameter set 4 epi-endo configuration. Details as in Fig 10. Amplitude of applied voltage is 2.3 kV.

The situation is markedly different for the epi-endo configuration. While the cross sections still have an orientation that follows the local fiber direction (Fig 13B and 13C), and some variability in lesion width is apparent in the side view (see Fig 13D), these effects are much smaller than for the penetrating electrodes configuration. The width variability ranges from 1.03 to 1.35 (compared to 1.3 to 3.3 for the penetrating electrodes configuration). The reason for this difference in the atria (but not the ventricles) is that the atrium is not as thick as the ventricle (4 mm vs 10 mm). As the tissue get thinner (keeping specific conductivities constant), there is less and less space for lateral currents to move out of the projections of the electrodes.

## Discussion

We have developed a model of electrical field distribution in cardiac tissue and shown that it serves to predict ablation volumes in for 4 mm wall thickness and weak anisotropy (*a* = 2) for the penetrating electrode configuration. Extrapolating from our model, we find that for higher anisotropy ratio, fiber rotation in the ventricles may make it more challenging to create ablations of a desirable geometry with penetrating electrodes, both for thicker tissues (ventricles) and thinner tissues (atria). With the epi-endo electrode configuration, however, our model predicts that lesions become more uniform in thicker tissues and that for thinner tissues, excellent uniformity can be achieved without consideration of the local fiber organization.

### Field strength needed for ablation

We found that if we assume a lethal field strength of 3 kV/cm, our model predicts the experimentally determined ablation volume well. It stands to reason that this critical field strength should depend on the pulse protocol, i.e. the number of pulses and the frequency with which they are applied. In particular, a larger number of pulses should reduce the critical field. We have collected experimental data for 20 pulses at 1 Hz (instead of 6 pulses at 3 Hz), and find that the critical field strength is reduced by approximately 10% [13].

The lethal field strength we observed is an order of magnitude lower than lethal field strength that have been reported for nanosecond ablation of tumors (with different pulse protocols), e.g. 40 kV/cm pulses to treat melanomas [32–34] or 35–68 kV/cm to treat hepatocellular carcinoma [35, 36].

### The role of anisotropy

For a low anisotropy ratio (*a* = 2), the anisotropy does not substantially affect the ablation outcome. This means that lesions of uniform width can be produced with either the penetrating electrodes configuration or the epi-endo configuration. The lesion thickness can be adjusted by electrode separation and field strength for the penetrating electrode configuration, and via the electrode diameter and the field strength for the epi-endo configuration.

For high anisotropy, we have shown that an anisotropy ratio around *a* = 10 poses challenges in tissues for which fiber direction changes significantly across the ventricular wall. While it is certainly possible to achieve transmural ablation, the cross section of the ablated volumes will vary substantially across the wall, mirroring the change of the fiber direction.

### Penetrating electrodes vs. endo-epi

The performance of both electrode configurations is similar if the anisotropy is low, fiber rotation is low, or thickness is high. In those situations, our model predicts that both configurations can create ablation volumes of consistent width. In the case of substantial fiber rotation over a wall that is thin (e.g. 3 mm, Fig 13), however, the epi-endo configuration has a clear advantage because it can still create an ablation volume with consistent cross section, while the penetrating electrodes configuration produces more variable cross sections. This case is particularly important because includes the human atria, in which the majority of all ablations are performed.

The epi-endo configuration becomes more restricted as the wall thickness increases. The field necessary to ensure transmurality increases with thickness, and therefore lesions have to be wider for thicker tissues in order to be transmural. For the penetrating electrodes configuration, on the other hand, the minimum field strength required depends on the electrode separation and is independent of tissue thickness, so thin, transmural lesions can be created even in thick tissue.

### Making lesions from individual ablations

If an individual ablation volume has a consistent cross section, it is simple to make lesions of uniform width. It is sufficient to move the electrodes along the desired ablation line, by a distance over which the width of the individual ablation volume does not change too much. If the ablated volume in good approximation rotationally symmetric, creating lesions becomes particularly convenient because there is no need to worry about the orientation of the ablated volume. If, on the other hand, there is substantial variation in the cross section of an individual ablation volume (see, e.g. Figs 10D, 11D and 12D), making lesions becomes more challenging. This problem is aggravated by the absence of detailed information on the fiber architecture in a practical setting and may require multiple ablations per site and make the outcome less predictable.

From these considerations, the epi-endo electrode configuration is very attractive for creating atrial lesions (e.g. to treat atrial fibrillation), because it creates approximately cylindrical ablation volumes, independent of the local fiber orientation. Generally, our results suggest that both penetrating electrodes configuration and epi-endo configuration should give excellent results in areas of low anisotropy ratio (*a* ≈ 2), but that variations in lesion width, as they are typical for RF ablation, should be expected for areas of high anisotropy ratio.

### Thermal effects

Our simulations show that thermal effects of pulsed electric field ablation with the shock parameters should be insignificant. Experiments confirm that no increase in temperature can be detected for the pulsing conditions that have been shown experimentally to lead to fully transmural ablation.

### Clinical implications

The consistent cross section of lesions made with the epi-endo configuration in thin preparations, independent of fiber rotation and anisotropy ratio, makes it an interesting candidate for reliable atrial ablations.

In the large part of the atria in which there is no substantial fiber rotation across the wall, both the epi-endo configuration and the penetrating electrode configurations can be expected to produce lesions with consistent width.

Apart from lesion geometry, an advantage of the epi-endo configuration is that it does not require the insertion of electrodes into the myocardium, while the penetrating electrode configuration does. At the same time, the penetrating electrode configuration is conceived for application with a single catheter (presumably from the endocardium), while the epi-endo configuration requires two catheters.

For both electrode configurations, the absence of thermal effects is good news, because it means that the complications in RF- or cryoablation that are related to thermal effects will not be a concern for them.

It is also important to consider the practicality of high voltage application in a clinical setting. We envision two applications of nsPEF technology: First, the application between the jaws of a surgical clamp, as it is currently done for RF ablation during open heart surgery, and later via a catheter as in current catheter ablation of RF. When used with a surgical clamp, there are no technical challenges beyond the general need of appropriate insulation and care when dealing with high voltages. Catheter delivery will indeed pose technical challenges, especially regarding the insulation of leads that are in close proximity.

### Limitations

We compute only stationary field distributions and use a continuous tissue models that is characterized by a variable conductivity tensor. This approach does not allow us to study the underlying mechanism of ablation, which involves cellular phenomena such as membrane permeabilization, and we are not able to predict the effect of varying ablation parameters (such as pulse number and duration). This approach does, however, allow the study of the lesion characteristics that can be achieved with different electrode configurations and the role of pulse amplitude.

The model we present here does not include tissue heterogeneities such as fibrous tissue or blood vessels. Both these omissions are justified to some degree by the fact that the model predictions coincide with experimental measurement.

Finally, our comparison between experimentally determined and theoretically predicted ablation volume is not a quantitative validation, because the staining technique can produce unstained tissue not only in ablated areas but also those that were not properly perfused; this creates some uncertainty about the extent of ablation.
